# Current scientific evidence for integrated community case management (iCCM) in Africa: Findings from the iCCM Evidence Symposium

**DOI:** 10.7189/jogh.04.020101

**Published:** 2014-12

**Authors:** Theresa Diaz, Samira Aboubaker, Mark Young

**Affiliations:** 1 Health Section, Programme Division, UNICEF, New York, NY, USA; 2 Adolescent and Child Health, World Health Organization, Geneva, Switzerland

## Abstract

In March 2014, over 400 individuals from 35 countries in sub-Saharan Africa and 59 international partner organizations gathered in Accra, Ghana for an integrated Community Case Management (iCCM) Evidence Review Symposium. The objective was 2-fold: first, to review the current state of the art of iCCM implementation and second, to assist African countries to integrate lessons learned and best practices presented during the symposium into their programmes. Based on the findings from the symposium this supplement includes a comprehensive set of articles that provide the latest evidence for improving iCCM programs and ways to better monitor and evaluate such programs.

Since early 2000 the use of integrated community case management (iCCM) strategy to deliver pneumonia, malaria and diarrhea treatments to children under 5 has dramatically increased. In 2005 there were only 10 countries in sub-Saharan Africa with policies supporting implementation of iCCM of which 7 included pneumonia treatment [1]. This increased to 28 countries by 2013 that now support implementation of iCCM and this includes pneumonia treatment [2].

iCCM, in the hands of well trained, supplied and supervised community health workers can reduce child mortality [3,4]. Recognizing this, in 2012 the World Health Organization and UNICEF released a Joint Statement for iCCM as an equity-focused strategy to improve access to case management, emphasizing important standard practices that should be part of any such programming in countries [5]. However, iCCM implementation has faced challenges considering the poor health care infrastructure in the countries in which this strategy has been introduced.

Since the joint statement was released an increasing amount of evidence has been generated on the strengths and limitations of iCCM, as well as the outcome and impact of iCCM within a variety of different country contexts [6]. A number of key impact and outcome studies were finalized in 2013. New and innovative methods for reporting and supervision have been tested. A comprehensive assessment of cost drivers and cost-effectiveness of this approach is ongoing. Finally, given the acceptance of iCCM as a delivery strategy to reach particularly those with limited access to health services, there has been much interest to add other interventions to the package such as maternal and newborn but these additions are only currently being tested in a few countries.

Between 3 and 5 March 2014, over 400 individuals from 35 countries in sub-Saharan Africa and 59 international partner organizations gathered in Accra, Ghana for an iCCM Evidence Review Symposium. The objective of the symposium was 2-fold: first, to review the current state of the art of iCCM implementation by bringing together researchers, donors, governments, implementers and partners to examine the current iCCM implementation landscape and status of evidence in key programme areas, in order to summarize lessons learned and best practices, and identify priorities and gaps in knowledge for improving maternal-newborn and child health. Second, to assist African countries to integrate lessons learned and best practices presented during the evidence symposium into their programmes and identify key actions to include in their national plans.

**Figure:**
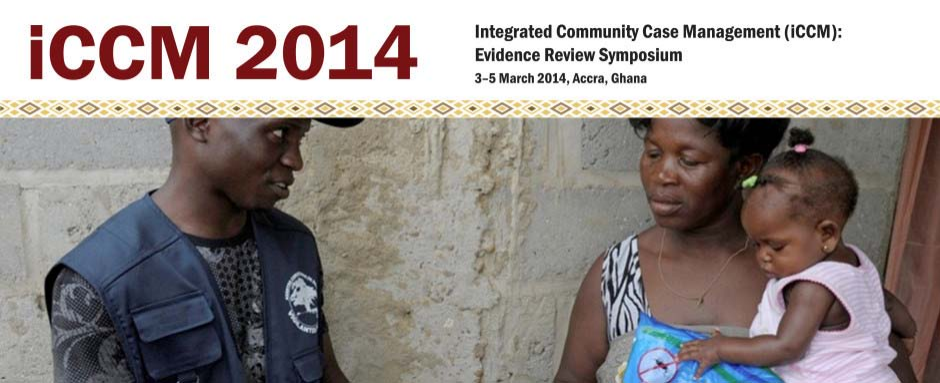
Photo: Courtesy of UNICEF

We conceptualized a theory of change model as to what factors may increase utilization of quality iCCM services (**Figure 1**). We assumed that this increase in utilization would translate into increased coverage and thus ultimately contribute to decreased childhood mortality.

**Figure 1:**
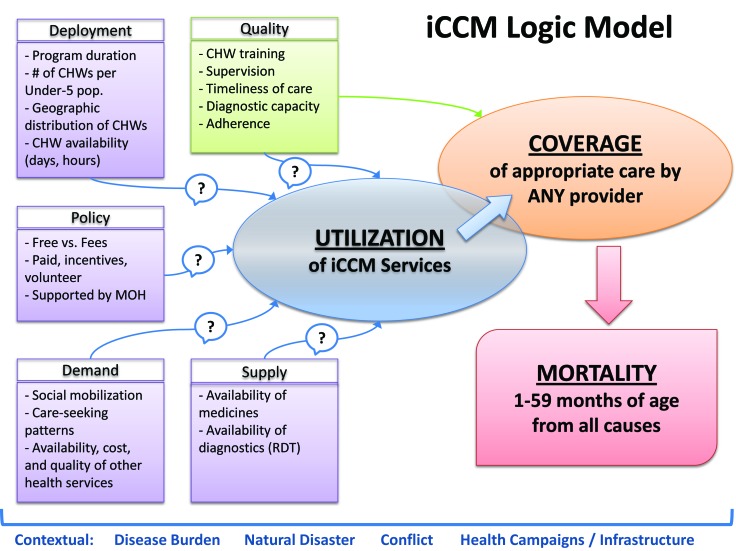
Logic model of integrated community case management.

The symposium and articles in this supplement were organized in thematic areas based on this model. Presentations were given in each thematic area and tools to support implementation were shared with participants for each of these areas as well as in three additional topic areas (private sector, innovations and newborn interventions). In addition lessons learned were documented in each area. This supplement includes articles, based in part on these experiences and lessons learned. The thematic and additional areas, their relationship to the model, and their associated articles are shown in **Box 1**.

Box 1 **Thematic areas, relationship to conceptual model and associated articles**

1. **Coordination, Policy Setting and Scale-up**: the current state of iCCM policies in Africa and challenges in development of policy and scale up– RELATES TO POLICY

Rasanathan et al. Community case management of childhood illness in Sub-Saharan Africa: Findings from a cross-sectional survey on policy and implementation (article # 020401)

Rasanathan et al. Where to from here? Policy and financing of integrated community case management of childhood illness (iCCM) in sub-Saharan Africa (article # 020304)

2. **Human Resources and Deployment**: community health worker (CHW) selection, geographic disbursement, motivation and retention – RELATES TO DEPLOYMENT

Pratt et al. Spatial distribution and deployment of community-based distributors implementing integrated community case management (iCCM): GIS mapping study in three South Sudan states (article # 020402)

3. **Supervision & Performance Quality Assurance**: strategies to ensure high quality care including strategies for effective training, use of alternative models for supervision, and the role of mHealth to support and motivate CHWs to provide quality care – RELATES TO QUALITY

Bosh-Capblanch and Marceau. Training, supervision and quality of care in integrated community case management (iCCM) programmes in sub-Saharan Africa (article #020403)

Strachan et al. The scale up of integrated community case management of malaria, pneumonia and diarrhoea across three African countries: A qualitative study exploring lessons learnt and implications for implementation (article # 020404)

4. **Supply Chain Management**: which systems ensure continuous supply, how best to forecast needs – RELATES TO SUPPLY

Chandani et al. Evidence for improving community health supply chains from Ethiopia, Malawi, and Rwanda (article # 0204005)

Sheishia et al. Strengthening community health supply chain performance through an integrated approach: Using mHealth technology and multilevel teams in Malawi (article # 020406)

5. **Costs, and cost-effectiveness and financing**: identifying cost drivers, improving cost-effectiveness and the importance of minimizing patient costs – RELATES TO POLICY AND DEMAND

Jarrah et al. The costs of integrated community case management (iCCM) programs: A multi-country analysis (article # 020407)

6. **Monitoring, Evaluation and Health Information Systems**: innovations in monitoring, integrating with health management information systems, using results to drive programmatic decision-making and improvements, evaluation design and methods – RELATES TO ALL AREAS

Guenther et al. Routine monitoring systems for integrated community case management programs: Lessons from 18 countries in sub–Saharan Africa (article # 020301)

Oliphant et al. Multi-country analysis of routine data from integrated community case management programs in sub-Saharan Africa (article # 020408)

Diaz et al. A proposed model to conduct process and outcome evaluations and implementation research of child health programs in Africa using integrated community case management as an example (article # 020409)

7. **Demand generation and social mobilisation**: the relationship between iCCM and care-seeking, treatment utilisation and treatment adherence, effective strategies to generate demand – RELATES TO DEMAND

Sharkey et al. Demand generation and social mobilisation for iCCM and child health: Lessons learned from successful programmes in Niger and Mozambique (article # 020410)

8. **Impact and outcome evaluations**: Issues with measuring mortality and using coverage to model mortality– RELATES TO COVERAGE AND MORTALITY

Amouzou et al. Assessing the impact of the integrated community case management (iCCM) programmes on child mortality: Review of early results and lessons learned in sub-Saharan Africa (article # 020411)

Friberg et al. Using the Lives Saved Tool as part of evaluations of community case management programs (article # 020412)

Additional topic areas:

Newborns – Aboubaker et al. Community health workers: a crucial role in newborn health and survival (article # 020303)

Research – Wazny et al. Setting global research priorities for integrated community case management (iCCM): Results from a CHNRI (Child Health and Nutrition Research Initiative) exercise (article # 020413)

Private sector – Awor et al. Integrated community case management and the private sector in africa – a relevant experiences and potential next steps (article # 020414)

Conclusions – Young et al. The way forward for integrated community case management programmes (article # 020304)

In regards to policy and scale up Rasanathan et al report on the results of survey on iCCM in sub-Saharan Africa and in a viewpoint summarizing policy issues that impact scale up and sustainability. In the area of human resources and deployment Pratt et al detail the use of GIS mapping in three states in Southern Sudan to improve deployment of CHWs. For training and quality Bosh-Capblanch et al provides a systematic review of training supervision and quality of care, while Stratchan et al present a qualitative assessment of implementation practices in 3 African countries. In regards to maintaining supply chains Chandani et al describe how three countries were able to ensure commodities and supplies reached community health workers, while Sheishia et al describe an innovative technology to track supplies in Malawi. To examine costs of iCCM, Jarrah et al describe the cost drivers of iCCM in multiple countries using standardized methods. In the area of monitoring and evaluation Guenther et al put forth components and attributes of a comprehensive monitoring system for iCCM, while Oliphant et al use routine monitoring from multiple country programs to demonstrate the utility of these data to examine key factors of program success or failure. Diaz et al review the study design and data elements collected for multiple recent evaluations of iCCM to suggest how such evaluations can be done in the future. In regards to demand, Sharkey et al present experience in two countries and the lessons learned on demand generation and social mobilization for iCCM. Finally, in the area of impact and outcome evaluation Agbessi et al report on the limitations of measuring and using mortality to assess iCCM as an end point in several countries in Sub-Sharan Africa and Friberg et al demonstrate how the Lives Saved Tool could be used to model the mortality impact of iCCM.

In addition to the articles covering the key thematic areas of the symposium we also have three additional articles. Awor et al describe how the private sector can contribute to iCCM. Aboubaker et al also present the role of Community Health Workers in newborn survival. Based on a systematic process to prioritize research areas, known as CHNRI (Child Health and Nutrition Research Initiative) Wazny et al report on priority iCCM research areas that are still needed. Finally, using the evidence available, Young et al suggest a way forward to improve and sustain iCCM where it is needed. This comprehensive set of articles provides the latest evidence for improving iCCM programs and ways to better monitor and evaluate such programs.
